# A Novel Helmet Fitness Evaluation Device Based on the Flexible Pressure Sensor Matrix †

**DOI:** 10.3390/s19183823

**Published:** 2019-09-04

**Authors:** Jianwei Niu, Cong Zhang, Xiao Chen, Chuang Ma, Liyang Chen, Chao Tong

**Affiliations:** 1 School of Mechanical Engineering, University of Science and Technology Beijing, Beijing 100083, China (C.Z.) (C.M.) (C.T.); 2 Military Institute of Engineering and Technology, Academy of Military Sciences, Beijing 100091, China; 3 Guanghua School of Management, Peking University, Beijing 100871, China

**Keywords:** flexible sensor, curved surface, sensor layout, head pressure, matrix network

## Abstract

Helmet comfort has always been important for the evaluation of infantry equipment accessories and has for decades not been well addressed. To evaluate the stability and comfort of the helmet, this paper proposes a novel type of helmet comfort measuring device. Conventional pressure measuring devices can measure the pressure of flat surfaces well, but they cannot accurately measure the pressure of curved structures with large curvatures. In this paper, a strain-resistive flexible sensor with a slice structure was used to form a matrix network containing more than a 100 sensors that fit the curved surface of the head well. Raw data were collected by the lower computer, and the original resistance value of the pressure was converted from analog to digital by the A/D conversion circuit that converts an analog signal into a digital signal. Then, the data were output to the data analysis and image display module of the upper computer. The complex curved surface of the head poses a challenge for the appropriate layout design of a head pressure measuring device. This study is expected to allow this intuitive and efficient technology to fit other wearable products, such as goggles, glasses, earphones and neck braces.

## 1. Introduction

With the increasing diversification of modern work and life, people are paying more attention to life safety [1]. The use of head protection products is becoming increasingly common as well; e.g., [2] head protection products are used in the military, physical work and sports. Wearing protective equipment can effectively protect the wearer’s physical safety but due to the special requirements of this equipment regarding performance, people must pursue a balance between protection ability and fitness [3].

At present, helmets are widely used in the military and medical, sports, entertainment and other industries. In addition, the methods to improve the safety and fitness of helmets have received extensive attention as well [4]. Helmet fitness performance refers to the match between the inner shape of the helmet shell and the outer shape of the head, the adaptability of the helmet’s accessory setting and the head shape distribution, the offset between the center of gravity of the helmet and its reasonableness to provide a pressure distribution on the head, and the matching of the suspension system to the head shape. Fitness performance is the indicator for the ergonomics, comfort and stability of the helmet.

To ensure the fitness of the helmet, people have performed in-depth studies on data acquisition, modeling and standardization using three-dimensional heads and detecting the physical properties of helmets [5], such as the center of mass, moment of inertia center of mass [2], head geometric feature description [4], dynamic response of the helmet biomechanical system to an impact force [6] and other characteristics. Among all the metrics related to the ergonomic design of the helmet, the pressure at each point where the helmet is in contact with the head is the most intuitive and efficient measure to evaluate the fitness performance of a helmet [7].

Cai used a Kinect sensor to scan a client’s head and compared the head shape to helmet shapes from a database consisting of off-the-shelf helmets [8]. The Kinect sensor is able to analyze the difference between the shape of the head and the helmet, indirectly obtain the pressure point and determine whether excessive pressure is generated. However, this method cannot directly obtain the pressure value, so it affects the accuracy and is overly cumbersome in implementation. If pressure can be directly measured when the helmet is in contact with the head, the utility and accuracy of the device will be greatly improved. Bibbo et al. studied the seat pressure sensing device. To better measure pressure when the human body touches a seat, they used the ATS (Analog Tactile Pressure sensors) method [9], and Hughes-Riley et al. performed a plantar pressure measurement that used a force-sensitive resistor device [10]. These methods can measure the plane pressure, but they cannot be used to measure the pressure of a helmet due to the complex curvature of the head. To measure the pressure on the head when protective equipment such as a helmet is worn, the pressure sensing device must be flexible. For a contact surface that approximates a plane, the traditional pressure sensor can measure pressure using the tile layout. However, for a curved structure with a large curvature variation, it is difficult for the traditional layout to obtain accurate and reliable data. Pressure sensors are primarily used to measure local pressure. Memarian applied pressure sensors to the comfort measurement used by the apparel industry [11]. In addition, sensors are used in the medical industry. Bootha used foot pressure to detect thumb valgus and sacral pain [12]. Heydaria placed a sensor on patients’ chest to make blood pressure measurements [13]. Therefore, a flexible sensor that can be used on a curvature surface with a novel sensor matrix was proposed in this study. Reports on the use of pressure sensing devices on curved surfaces are rare. Parmar et al. studied the effects of flexible sensors in the treatment of chronic application [14] and found that curvature and stiffness changes of the substrate hardly affected the performance of the sensors [15]. Because of the high flexibility of sensors, their reliability is 80% [16] and their sampling frequency is high (up to 10 kHz). In this study, a pressure sensor developed by i-motion [17] is used in our helmet pressure sensing device.

The subjective evaluation method has long been used to evaluate the stability and comfort of the helmet. The tedious evaluation method has a long information acquisition period and a high experimental workload. It is difficult to obtain quantitative conclusions, and many human error factors affect the data. The measurement system used in this study is more objective and quantitative than that used in the subjective evaluation method. The measurement system used in this study can test the mechanical or kinematic indicators of the helmet in real time. In addition, software was developed to facilitate a follow-up data analysis and evaluation of the helmet fitness to optimize the ergonomic performance the helmet.

## 2. Helmet Fitness Measuring Device

**Figure 1:**
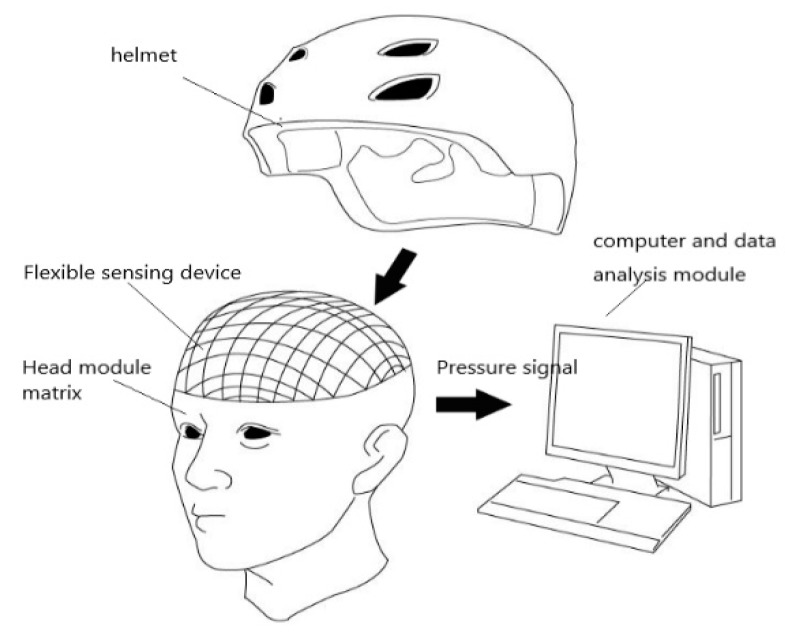
Overall functional design of the system.

The device used in this study is a testing platform to study the ergonomics and mechanical properties of helmets based on a pressure measurement, including signal acquisition (pressure sensor), data integration and processing (lower position machine), and data processing and display (host computer) as shown in Figure 1. The signal acquisition module is responsible for measuring pressure data, controlling the multiplexing and amplifying the data signals of the varistor. The data processing module is responsible for converting the analog sensor signal into a digital signal, i.e., A/D conversion; performing data integration and processing; and transmitting sensor data to the computer. The data display module is responsible for accepting the data from the lower computer, filtering the signal noise with the receiving time as the axis, drawing the three-dimensional image (head surface), and projecting the three-dimensional image to the display window according to the selected mode.

### 2.1. Standard Human Head Modeling

**Figure 2:**
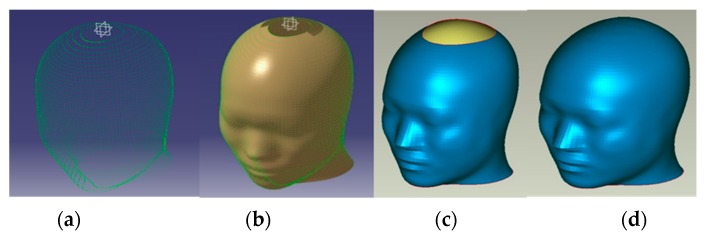
Head model reconstruction procedure: (**a**) point cloud; (**b**) layered scan; (**c**) hierarchical modeling; (**d**) head model.

The helmet testing system we built is based on the Chinese standard head type for measuring helmet fitness. We developed a standard head model which conformed to the Chinese head type. The pressure head cap was designed according to the standard head model. This paper used reverse modeling technology, which can effectively overcome the shortcomings of the traditional 3D scanning method [18] and anatomy-based model construction method [19]. Reverse modeling technology has a lower cost and stronger universality than other methods as well. In addition, the concept of free shape deformation (FFD) [20] of digital sculpture tools in computer aided design (CAD) is used. This paper proposed a new method of head modeling based on an unorganized point cloud. This method can quickly establish a standard head model that conforms to the human body standard. As shown in Figure 2, we first obtained the head point cloud using laser layered scanning technology. The scanned samples were Chinese males, and the samples were ethnically and regionally representative. Then, the point cloud was imported into CATIA(Dassault Systèmes’ 3D PLM product) for materialization and ZBRUSH, a digital sculpting tool that combines 3D/2.5D modeling, texturing and painting, to smoothen the model, and a standard head type outer layer was constructed. We obtained 44 different typical head models and used UGNX, an advanced high-end CAD/CAM/CAE owned since 2007 by Siemens PLM Software, to make head circumference measurements on the standard head model. Using the digital engraving technique to construct the head model, four different standard solid head models which cover most Chinese male head shapes and features were built. In this study, four silicone pressure head sleeves that matched the head model were constructed, which greatly improved the flexibility of the device.

### 2.2. Pressure Test Sub-System

The sensor uses a flexible film grid sensor from I-motion, China, and the sensor model number was IMS004-C20B. I-motion sensors can be made with different shapes, sizes and spatial resolutions, and the pressure measurement range of these sensors is from 0–50 g/cm^2^ to 0–100 kg/cm^2^. Based on the dimensional characteristics of the test piece, I-motion offers the best match, spatial resolution and pressure range.

**Figure 3:**
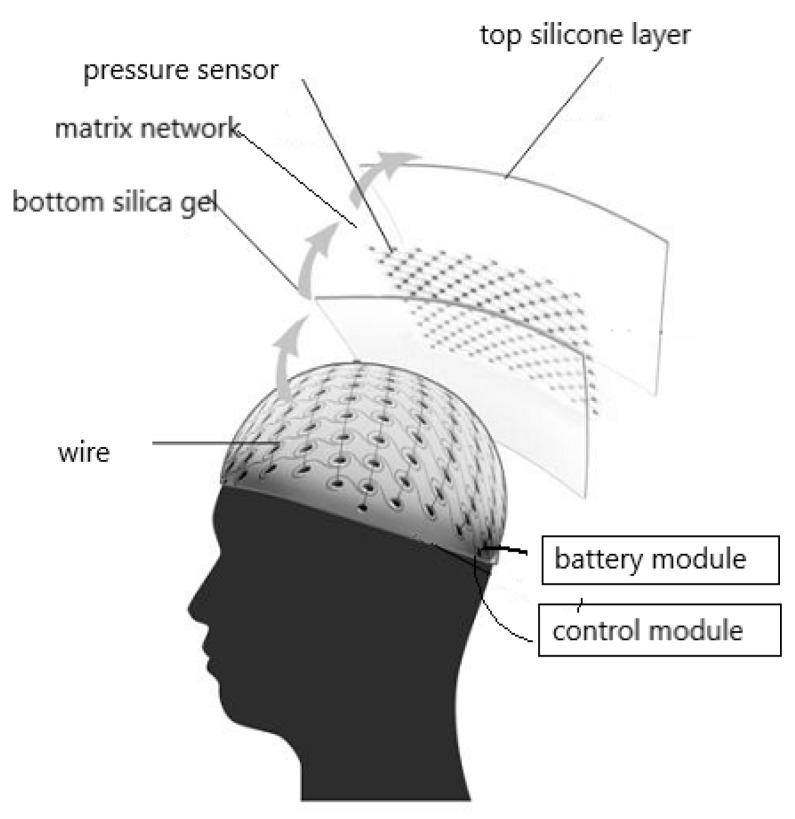
Schematic of the data acquisition system.

The device comprises a bottom silica gel layer which closely conforms to the shape of the curved surface of the head. A matrix network consisting of many pressure sensors and wires is laid out on the upper surface of the bottom surface silicone layer. The power module and acquisition control module are connected by wires outside the matrix network. A top surface silicone layer is placed on the matrix network and bottom silicon layer to bond the bottom silicone layer, pressure sensor and wires into a whole to form a flexible sensing device, as shown in Figure 3. The silica gel layer in the device is less than 0.25 mm thick, and the entire head cover is 0.5 to 0.7 mm thick. The silicone layer of the headgear is bound to the sensor layer. To prevent slippage and friction between the layers, the silica gel layer and sensor layer are joined by dot bonding. The silica gel layers are joined by strip bonding. This connection can avoid the disadvantages of increased thickness and reduced flexibility.

To facilitate the construction of the helmet pressure test system, we built a Chinese standard head model according to the national standard GB/T23461-2009, “three-dimensional size of adult male heads” [21]. Based on the outer surface of the Chinese standard head model, the size of the silicone layer and the sensor distribution were designed. Through data acquisition and analysis of the host computer, the system was able to measure data and evaluate helmet fitness.

#### 2.2.1. Circuit Design

The pressure acquisition module is composed of a flexible chip pressure sensor, a matrix scanning circuit, a resistance-voltage conversion circuit, and an analog-to-digital converter.

**Figure 4:**
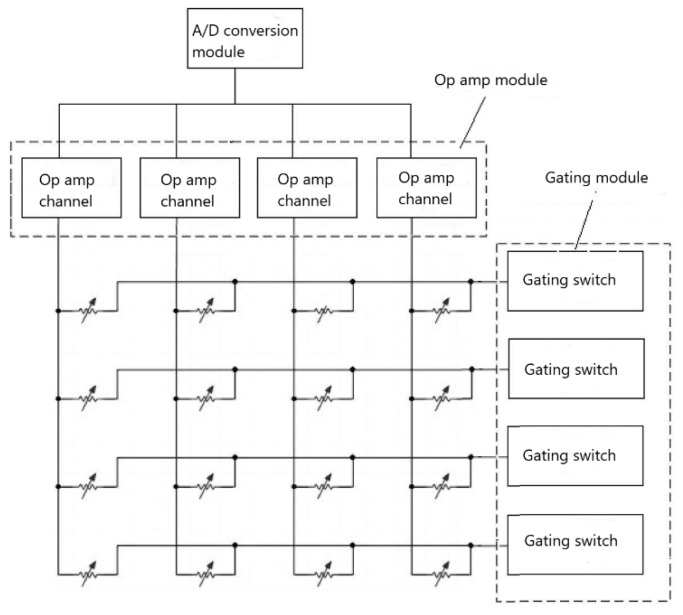
Schematic diagram of the arrangement of the pressure sensor elements (here, 4 × 4 is used to indicate the 16 × 16 original circuit).

The flexible sheet pressure sensor collects pressure information by using the change in resistance caused by the deformation of the piezoelectric rubber by pressure. When the pressure is large, the piezoelectric rubber is crushed, the distance between the two electrodes becomes small, and the electric resistance decreases. Due to the large number of sensors and low sampling frequency requirements, the sensor array is organized in a stereo matrix: the sensor consists of 16 rows, 16 columns, and four blocks. A schematic diagram of the arrangement of the pressure sensor elements is shown in Figure 4. Among them, 16 lines are directly connected with 16 amplifying circuits; the resistance signal is converted into a voltage signal and sent to the 16 built-in analog-to-digital converter pins in the single-chip microcomputer. The collected data are in parallel, and the sampling precision is 8 bits per sample. When a pressure sensor is strobed, the corresponding channel’s op-amp channel collects and amplifies the current sensed by the pressure sensor. The output is obtained using the A/D conversion module. Thus, the outputs for all of the pressure sensor currents are collected. The program control microcontroller scans five times per second and obtains pressure information of 5 × 16 × 16 × 4 bytes.

**Figure 5:**
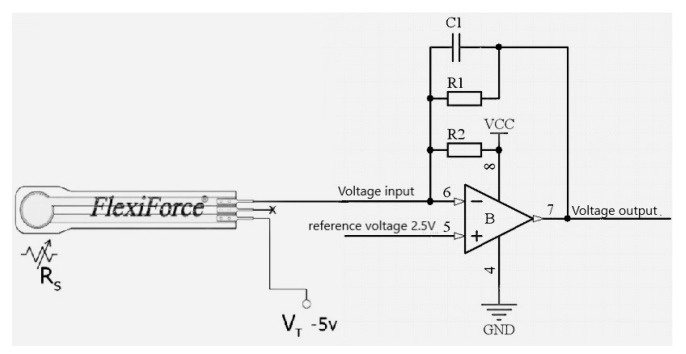
Data acquisition circuit.

The resistance-voltage conversion circuit is composed of four A/D conversion modules, and each A/D conversion module integrates four operational amplifier channels. The circuit is shown in Figure 5. C1 is responsible for low-pass filtering used to eliminate interference and jitter. R1 sets the conversion sensitivity, and R2 sets the initial voltage value. The voltage applied to the sensor is always 2.5 V.

#### 2.2.2. Pressure Sensor Layout Design

A reasonable layout can effectively improve the reliability of the sensor [21]. To uniformly sample the pressure points, we arranged the sensors using the conic equidistance method. The entire sensor arrangement is shown in Figure 5. The original construction data of the head model is a cylindrical coordinate system, i.e., it is divided into several layers at equal distances according to height (Z axis) and 120 coordinate points are picked from each layer. One coordinate point is taken every 3°.

Then, we constructed the surface of the head model in the coordinate system of Siemens U using the classic CAD (Computer Aided Design) software. Then, the pressure test area (i.e., the head cover range) was cut out with a plane. The intersection of the plane and head surface was approximately circular, so we constructed a circle similar to the curve and used Team Viewer to obtain the coordinates of the center of the circle as the center of the new coordinate system.

The Z coordinate axis of the new coordinate system was perpendicular to the tangent plane. Taking the new Z coordinate axis as the base coordinate axis and the new coordinate point as the apex, we constructed eight conical surfaces which intersected the head model surface to obtain eight intersecting lines. With the intersecting line between the cutting plane and the head model surface, nine closed curves were obtained in total. Four sensor elements are arranged at equal intervals on the topmost circle, and the sensors are arranged at the intersection of the lowest tangential planes, according to the principle of increasing the difference of 4°. We named this method the Conic Equidistance Method (CEM).

**Figure 6:**
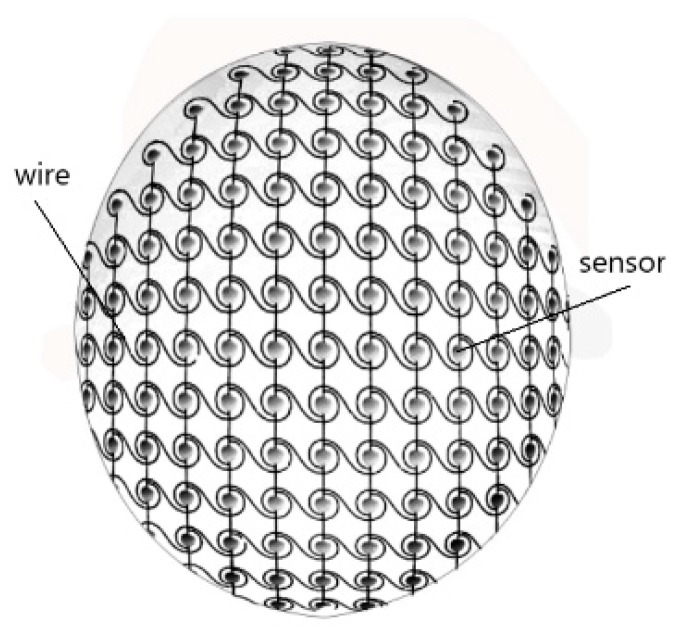
Sensor and wire layout using the Conic Equidistance Method.

As shown in Figure 6, the sensors are spread over the pressure points of the head. Moreover, the bottom layer of the silica gel and the top surface silica gel are arranged on the upper and lower layers of the sensor, which provides a uniform surface for the pressure sensor array and functions as an electrical insulation to ensure the stable operation of the pressure sensor array. In addition, the pressure sensors between the matrix networks are welded to the coiled curve, so the device has good ductility. The pressure sensors can simultaneously measure the current from any row or any column. The efficiency of the device is very fast, and the sensors do not interfere with one another.

In the process of testing the helmet pressure with the headgear, the headgear device was bonded by a two-layer silicone layer and a layer of pressure sensors. During testing, the participant wears the headgear, which fits the wearer’s head curve well during the experiment. To ensure the practicability of the headgear, we tailored the silicone layer according to the standard head model, as described in Section 2.1, to design a headgear which conformed to the characteristics of the head. According to different shape characteristics of the head, we designed five headgears based on the helmet sizing systems defined by the GJB 5691-2006 (Chinese)-sizing system of military helmets.

### 2.3. Lower Machine Sub-System

The lower computer is mainly responsible for data processing. It can convert the analog signals from the sensor into digital signals for data integration, processing, and transmission to the computer. The lower computer mainly includes a single-chip microcomputer and a wireless Bluetooth transmission module.

#### 2.3.1. Single Chip Microcomputer

For this device, a MC9S12XS128 single-chip microcomputer was selected. This single-chip microcomputer has the advantages of error correction capability, good flexible customization mode, background debugging mode and compression code fully optimized for C language.

In the development of the microcontroller, the platform was built by CodeWarrior. The constructed lower computer platform can convert, integrate and transmit data from the pressure sensors. The sensor consists of 16 rows, 16 columns, and four blocks. The four blocks are controlled by four IO ports, and only one block is simultaneously strobed. The 16 columns are controlled by 16 pins of the single-chip PA0~PA7 and PB0~PB7 ports, and only one column is simultaneously strobed. The 16 lines are directly connected to the 16 amplifying circuits, and the resistance signals are converted into voltage signals and sent to the 16 built-in analog-to-digital converter pins AD0~AD15 in the Micro Controller Unit (MCU). Thus, each pressure sensor unit corresponds to a unique matrix ID. The program control microcontroller scans five times per second and collects pressure data of 5 × 16 × 16 × 4 bytes.

#### 2.3.2. Bluetooth Transmission Device

To complete the transmission of data between the upper computer and lower computer and to enhance the usability of the device, we changed the traditional wired transmission to Bluetooth transmission. Bluetooth transmission is more convenient and efficient for dynamic data measurements. We used a wireless Bluetooth configuration on the host computer and the lower computer.

**Figure 7:**
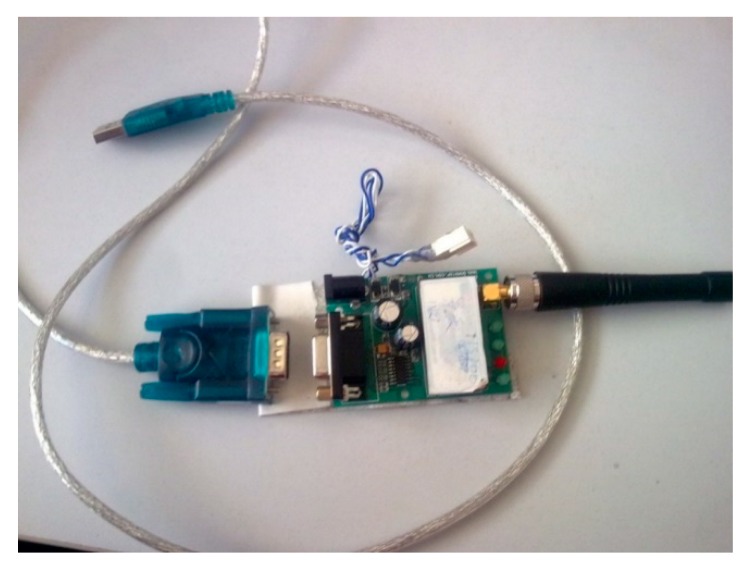
Wireless communication module and computer connection.

The MC9S12XS128 MCU uses the Serial Communication Interface (SCI) protocol. By controlling different registers, the data transmission is realized. As shown in Figure 7, the green circuit board and white wireless module have a 5-V power supply interface and a serial port. The green head silver line is the “USB-serial” (a Universal Serial Bus) data conversion line. The serial port is plugged into the wireless module driver board, the USB port is connected to the PC (Personal Computer), and the driver of the “USB-serial port” line is installed on the PC.

### 2.4. Host Computer Sub-System

**Figure 8:**
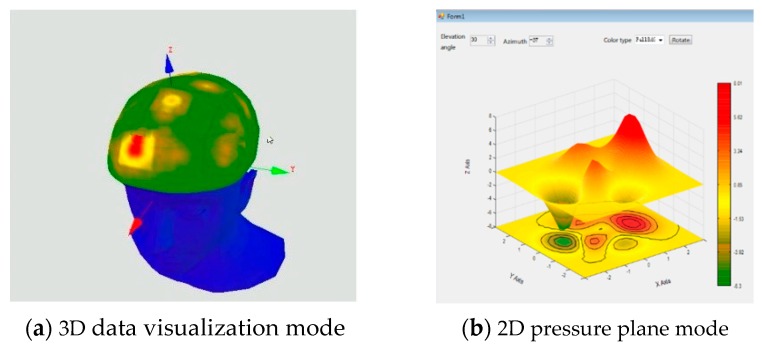
Image generation.

**Figure 9:**
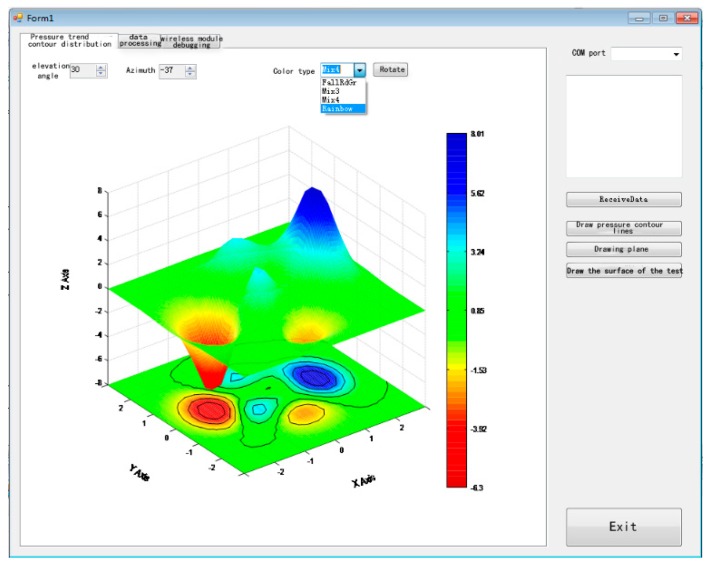
Host computer software interface.

The host computer includes a data processing module, a drawing and display module, and a helmet ergonomic evaluation module. The functions of this program are mainly to receive data, denoise and process the data, and finally, generate a visual pressure image. According to different requirements, the program can generate a 3D data visualization or 2D pressure plane model, as shown in Figure 8 and Figure 9.

The development of the host computer program is based on the Microsoft Visual Studio 2008 platform, and the programming language is C#. The main fitness metrics are the helmet pressure center, helmet pressure resultant vector, helmet standard pressure difference and other data. After generation of the pressure distribution cloud map, the fitness of the helmet is evaluated according to the standardized pressure value and a subjective evaluation method. The subjects were asked to provide subjective score from two categories, i.e., pressure and stability. Taking pressure as an example, the comfort evaluation was divided into eight categories: more comfortable, a bit comfortable, average, a bit uncomfortable, less comfortable, very uncomfortable, severe discomfort, and extremely uncomfortable, which corresponded to 0~2 kPa, 3~4 kPa, 5~6 kPa, 7~9 kPa, 10~13 kPa, 14~18 kPa, 19~25 kPa and 26~33 kPa, respectively. These categories are based on the expertise and comments of the helmet wearers. The collected pressure values are standardized and graded in three levels, and the results are compared with the questionnaire survey results to complete the comfort evaluation.

## 3. Helmet Fitness Evaluation Test

To test the accuracy of the device, we sampled data for a certain type of helmet. Eight subjects were recruited to wear the helmet when completing three tasks, and the subjective scoring results were tested for consistency with the evaluation results based on the objective pressure method. The eight participants were adult males aged 16–36 years and in good health. All subjects gave their informed consent for inclusion before they participated in the study and the protocol was approved by the Ethics Committee of University of Science and Technology Beijing. The model heads were screened, and eight representative subjects were obtained based on the GJB 5691-2006 (Chinese)-sizing system of military helmets, which defines the representative head models that cover most Chinese young males.

### 3.1. Device Calibration

Three levels of calibration were completed before the helmet was placed on a participant.

First, the sensor supplier provides the calibration results, i.e., the linear relationship between the loaded force and the conductance of the original single sensor. However, we cannot directly adopt their linear relationship because the assembly, headgear material and shape and size of the participant’s head affect the original calibration results.

**Figure 10:**
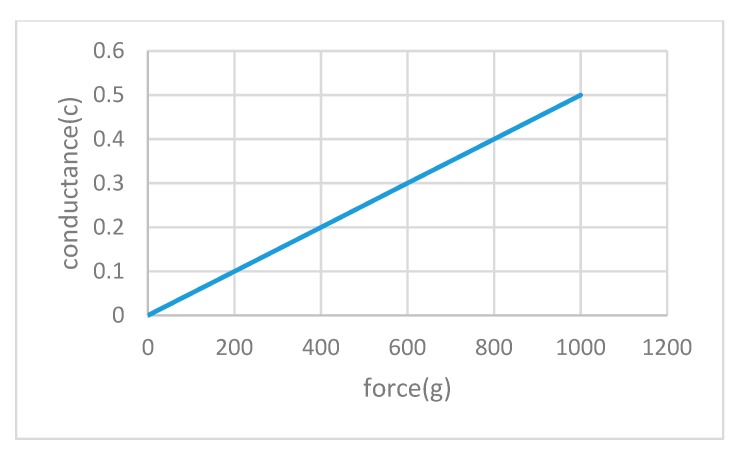
Linear relationship between the force and conductance of the bonded sensor.

Second, to avoid potential output deviations due to the device assemble procedure, we calibrated the sensors in the headgear before it was placed onto the head model. We randomly selected several sensors in the headgear, loaded a standard weight, measured the voltage output of the sensor circuit, changed the load and measured the voltage output of the sensor circuit again. Using this iterative procedure, we establish a linear relationship between two variables: force/pressure and the conductance. In detail, we use a weight (50 g, 100 g, 200 g, 500 g and 1000 g) as the standard load. When the sensor was unloaded, its resistance was very large and reached several tens of megaohms. When an external force was applied to the sensing area of the sensor, the resistance rapidly and significantly decreased—i.e., the resistance inversely changed with the change in external force, indicating that a higher pressure denotes a smaller resistance. We add a standard weight, change the load, measure the voltage output of the sensor circuit, repeat this procedure iteratively, and establish a linear relationship between the two variables—namely, force/pressure and conductance. The sensing area was 5 mm in diameter, so a weighted chassis was made with a diameter of approximately 3.5 mm and a weight of 1.35 g. The conductivity diagram produced after the test is shown in Figure 10 (C (conductance) = 1/R (resistance)); as observed from the curve, the conductance is linear with the force/pressure. After calibration, the stability of the repetitive test was verified. After the calibration, a linear relationship between the sensor load and the output voltage must be established to be displayed by the host computer. In the calibration process, we used the Chinese standard head model which we established to calibrate the head model. This calibration was more stable and eliminated the effects of many human instability factors. Because there are few similar products on the market, no comparison to similar products is made in this article.

Third, flexing to fit the shape and size of the participant’s head results in an output even before the helmet is added, i.e., the initial pressure or original pressure. Consequently, for in data processing, we consider the initial pressure for each sensor and conduct a zero calibration before the device is placed onto the participant’s head.

### 3.2. Helmet Fitness Data Analysis

We selected three tasks: walking, running, and kneeling forward. While the subject was wearing the helmet, the contact surface between the head and the helmet was divided into five zones: front, back, middle, left, and right. We collected the head force data, helmet stability data, and pressure center offset data during the three tasks.

**Figure 11:**
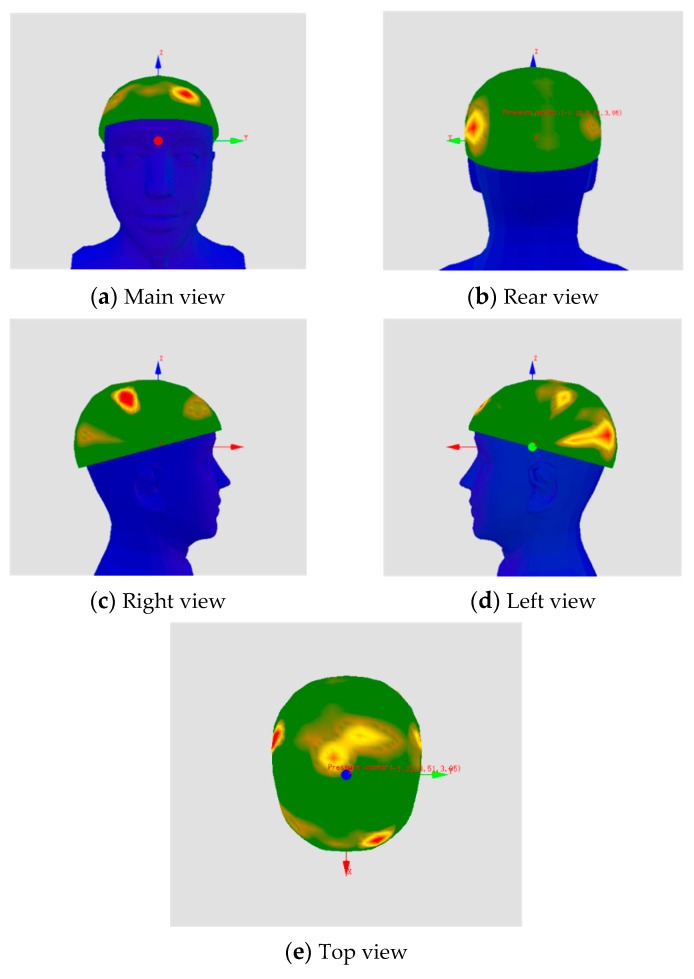
Helmet pressure cloud of one subject.

**Figure 12:**
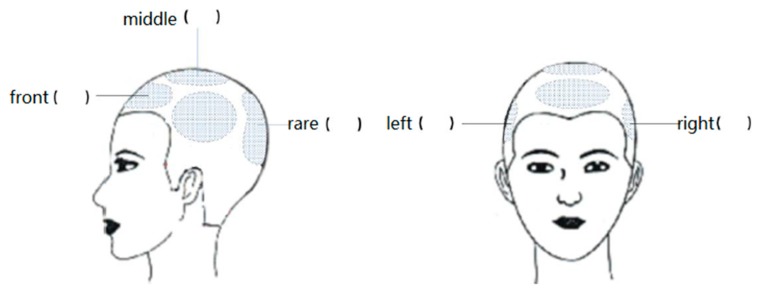
Head area division.

(1) The data collected by the host computer are shown in Figure 11. These data include the average pressure, peak pressure, average pressure value in the subarea, peak pressure in the subarea, and the impulse of the force (pressure × time). The test data are shown in Table 1 for eight subjects (Table 1 includes both measurement data and score data).

(2) For the stress situation, the subjects were asked to evaluate the comfort of the five areas of the helmet: front, middle, rear, left and right, as shown in Figure 12. The participants had to score the comfort of the different parts of the helmet in three states: upright, walking and running. The data shown in Table 1 are the data acquired by the host computer and the scoring of the helmet by the host computer. Three categories of indices, i.e., time, pressure and stability, were adopted in this model. Specifically, the time index consists of pressure impulse and pressure stability, while the pressure index corresponds to the average pressure and the historical peak pressure. As for the stability index, it comprises the pressure center movement range and the buffer capacity of the helmet. Finally, the analytic hierarchy process (AHP) was used to compute the weight coefficients of the indices to construct the fitness evaluation model. From the collected questionnaire, individual scores were summarized according to the evaluation requirements used to generate a summary table, as shown in Table 2. The questionnaire collected information on the helmet in three aspects: helmet comfort, stress situation, and stability score, and generated scores. To facilitate the study, before comparing the objective pressure value with the subjective results, we summarized the questionnaire results and integrated them into the items that we directly required. In addition, the subject was required to describe an uncomfortable area according to the different states and the direction of the center of gravity shift. Finally, the questionnaire survey results and measurement data were summarized, as shown in Table 3.

(3) From the output data from the upper computer presented in Table 1, we can determine the comfort of the helmet. The average head pressure was 0.52 Kpa, which is much lower than the critical threshold of 9.305 KPa for long-term wear, so the helmet will not cause serious pressure damage to the wearer. However, the average peak pressure of the entire head was 18.07 KPa, which exceeds the long-term wearing threshold of 9.305 KPa, so wearing this helmet for a long time may cause local physical discomfort. The average score was 69 and the comfort was medium, but the pressure distribution was uneven and not suitable for long-term wear—so the helmet should be improved.

### 3.3. Consistency Statistics

To verify the reliability of the device, we conducted a subjective questionnaire for the eight subjects to evaluate the subjective feelings of the subjects while walking, running, and kneeling forward. The Likert scale was used for the stability questionnaire and categorized the comfort level as: very uncomfortable (1), less comfortable (2), a little uncomfortable (3), normal (4), somewhat comfortable (5), more comfortable (6), and very comfortable (7). In Table 3, the performance test for the helmet is divided into a stress test and a stability test. Consistency tests were conducted from the subjective questionnaire and the objective test. The consistency of the subjective questionnaire and objective measurement data was 83.33% for pressure and 75.00% for stability, as shown in Table 3. Thus, the helmet fitness test system has strong credibility.

We observed that the helmet requires improvement based on the score from the upper computer and the consistency test results—which had an average score of 69 points. The test system evaluates the comfort of the helmet and provides a theoretical basis for improving the helmet. For example, the pressure cloud diagram from the upper computer shows that the force of the head can be obtained from Table 4 and that the peak area of the force is mainly at the front and left side of the head—which may be caused by a shift in the center of gravity or excessive parts on the front of the helmet. Consequently, we can focus on improving these two areas when improving the helmet type.

## 4. Discussion and Conclusions

The consistency test and experimental results show that the device has good precision and practicability. The device was constructed using a parametric 3D standard head surface creation method that was developed based on digital human body cross-section slice image data. In addition, this study shows that although the flexible sensor is not suitable for curved surface pressure measurements on heads with large curvatures, the head pressure can be measured well by the flexible sensor because the regularity of the human head and the layout density of the flexible sensor of our device are sufficiently large. The experimental results show as well that the multisensor assembly can eliminate the instability of a single sensor and improve the reliability of the device.

We proposed a novel device to objectively and quantitatively evaluate the stability and comfort of helmets, and we provided important quantitative indicators for the design and optimization of various helmets. The device has good flexibility and can closely fit the curvature of the head. Therefore, the device is particularly suitable for measuring the pressure on the head when wearing protective equipment—such as a helmet. The generation of the device provides a good detection method to determine the helmet fitness, and this method can be widely applied to military, vehicle, medical and other fields.

However, there are some limitations of this study: (1) although our equipment has certain flexibility, it has at present only been tested on young Chinese males; (2) our method is relatively novel, but the adaptability of helmets lacks international standards. Whether the selected indicators are reasonable and comprehensive and whether our Likert classification conforms to the actual situation requires further study. (3) In the future, the shear force between the inner liner of the helmet and the scalp or hair of the head should be considered. These studies require the joint efforts of domestic and foreign counterparts. (4) Although the sensor is flexible—it has inevitable defects. Since the head sensor is very thin, it is necessary to avoid strong tearing and sharp object collisions during production. In addition, the internal circuit of the flexible sensor is complicated and relatively small, so the flexible sensor can be easily broken. If the circuit inside the sensor is broken, it is difficult to repair. (5) To improve its usability, the device adopts wireless transmission technology, which has high bandwidth requirements. The wireless transmission of the control circuit box is similar to a tail hanging behind the head or attached to the helmet—which affects its usability.

## Figures and Tables

**Table 1:** Pressure test data and helmet score in the host computer.

ID	Average Pressure (Kpa)	Peak Pressure (Kpa)	Sub-Regional^†^ Average Pressure (Kpa)	Sub-Regional^†^ Peak Pressure (Kpa)	Impulse (Kpa/s)	Score			
						Time	Pressure	Stability	Total Score
1	0.36	21.66	0.21	6.84	4.07	82	30	93	71
			0.13	11.74					
			0.48	1.35					
			0.27	2.22					
			0.07	21.66					
2	0.4	14.89	0.2	14.89	3.9	83	30	94	72
			0.2	10.66					
			0.95	1.67					
			0.35	1.2					
			0.1	6.66					
3	0.76	10.89	0.28	9.58	7.96	77	27	93	69
			0.13	10.89					
			1.3	2.64					
			0.63	5.87					
			1.28	9.61					
4	0.75	20.75	0.29	1.52	8.32	78	27	88	67
			0.25	20.75					
			0.16	0.32					
			0.59	1.52					
			0	1.12					
5	0.69	17.74	0.38	17.74	5.57	79	27	85	66
			0.26	1.2					
			0.6	1.18					
			1.73	5.54					
			0.46	3.73					
6	0.27	18.83	0.13	11.69	2.89	83	31	95	73
			0	1.12					
			0.38	2.93					
			0.11	18.83					
			0.2	1.52					
7	0.37	24.09	0.38	24.09	4.47	77	30	84	66
			0	7.66					
			0.23	1.72					
			1.62	3.41					
			0.32	9.59					
8	0.59	15.77	0.33	15.77	5.72	77	28	89	68
			0.33	2.54					
			0.46	4.2					
			1.31	6.25					
			0.18	11.41					
AVG	0.52	18.07	0.432	7.37	5.36	79.5	28.75	90.12	69

^†^ Sub-region: front, back, left, right, middle.

**Table 2:** Summary of the helmet comfort questionnaire.

Item		Score
Helmet comfort		Very uncomfortable
Stress situation	Walk	The front and rear are not comfortable
	Run	The front, middle and left are very uncomfortable
	Kneeling forward	The front and rear are not comfortable
Follow stability comprehensive score		More unstable
the center of gravity shift		Focus on the back

**Table 3:** Consistency statistics of the pressure and stability of the helmet.

ID	Status	Pressure Related			Stability Related		
		Subjective Questionnaire	Measured Data	C	Subjective Questionnaire	Measured Data	C
1	Walk	F/B	F/B	Y	1	P	Y
	Run	F/M/L	M/B	N	2	M	Y
	Kneeling forward	F/B	F/B/L	Y	4	G	N
2	Walk	L/R	L/B	Y	4	M	Y
	Run	L/R	L/B	Y	4	P	N
	Kneeling forward	L/R	L/B	Y	4	P	N
3	Walk	F/L	F	Y	4	P	N
	Run	F/L/R	F/L	Y	3	P	Y
	Kneeling forward	F/R/L	F/R	Y	2	P	N
4	Walk	F/L/R	F/L/B	Y	3	P	Y
	Run	L/R	F/R	Y	4	P	Y
	Kneeling forward	F/R	F/B/L	Y	3	P	Y
5	Walk	F/L/R	F/R	Y	2	M	Y
	Run	F	F/L	Y	2	F	Y
	Kneeling forward	F/L/R	F/L	Y	2	F	Y
6	Walk	L/R	L/R	Y	7	M	Y
	Run	L/R	F	N	6	M	N
	Kneeling forward	L/R	F/L	Y	5	G	Y
7	Walk	L/R	F/L	N	2	P	Y
	Run	L/R	L/R	Y	2	P	Y
	Kneeling forward	L/R	F/L	Y	2	P	Y
8	Walk	F	F/B	Y	2	P	Y
	Run	L/R	F/L	N	3	P	Y
	Kneeling forward	R	F/R	Y	2	P	Y
C	83.33%				75.00%		

Abbreviations: front (F), back (B), left (L), right (R), medium (M), yes (Y), no (N), good (G), medium (M), poor (P), failed (F), consistency(C).

**Table 4:** Display from the host computer for the pressure peak area.

	Head Area	Front	Medium	Back	Left	Right
Status						
Walk		75.00%	0.00%	50.00%	50.00%	25.00%
Run		75.00%	12.50%	25.00%	50.00%	25.00%
Kneeling forward		87.50%	0.00%	37.50%	75.00%	25.00%
Percentage average		79.00%	4.00%	38.00%	58.00%	25.00%
